# Burkitt's Lymphoma Located in the Orbit: A Case Report

**DOI:** 10.7759/cureus.93995

**Published:** 2025-10-07

**Authors:** Boubacar Moussa Diadie, Ayoub Bakhil, Papys Mendes, Jalal Hamama, Karim El Khatib

**Affiliations:** 1 Stomatology and Maxillofacial Surgery, Mohammed V Teaching Armed Forces Hospital, Rabat, MAR; 2 Stomatology and Maxillofacial Surgery, Faculty of Medicine and Pharmacy, Mohammed V University, Rabat, MAR; 3 Anatomy and Pathology, Mohammed V Teaching Armed Forces Hospital, Rabat, MAR; 4 Anatomy and Pathology, Faculty of Medicine and Pharmacy, Mohammed V University, Rabat, MAR

**Keywords:** burkitt's lymphoma, diagnosis, high grade of malignancy, orbital location, polychemotherapy

## Abstract

Burkitt's lymphoma (BL) is an aggressive, fast-growing type of non-Hodgkin lymphoma that originates in B lymphocytes; it is more common in human immunodeficiency virus (HIV)-infected patients. BL is considered an AIDS-defining cancer. A young patient was being treated for HIV infection with orbital localization mimicking the clinical picture of orbital cellulitis. The patient underwent paracanthal surgical exploration of the left orbital cavity and an endosinusal biopsy, which confirmed a diagnosis of BL. Ocular involvement is extremely rare, but its presence in malignant hematological disorders may be indicative of the disease, requiring, in this case, an assessment of its extent to guide treatment and follow-up examinations. The histopathological diagnosis of BL is based on a biopsy of a lymph node or organ that may be affected. Polychemotherapy is the best treatment option. Although rare, BL is a highly malignant cancer. It is a medical emergency requiring polychemotherapy.

## Introduction

Burkitt's lymphoma (BL) is an aggressive, fast-growing type of non-Hodgkin lymphoma (NHL) that originates in B lymphocytes [1]. It can appear in the lymph nodes, as well as in organs or tissues other than the lymph nodes (extraganglionic sites). BL can affect adults, but it is more common in children and young adults. In human immunodeficiency virus (HIV)-infected patients, BL is considered an AIDS-defining cancer [1]. We report the case of a young patient being treated for HIV infection with orbital localization mimicking the clinical picture of orbital cellulitis.

## Case presentation

A 38-year-old patient, followed for HIV infection and under treatment for five years, presented to our emergency department for treatment of left orbital exophthalmos. The history of the disease dates back to seven days before his admission, with the onset of left exophthalmos with progressive protrusion of the eyeball.

**Figure 1:**
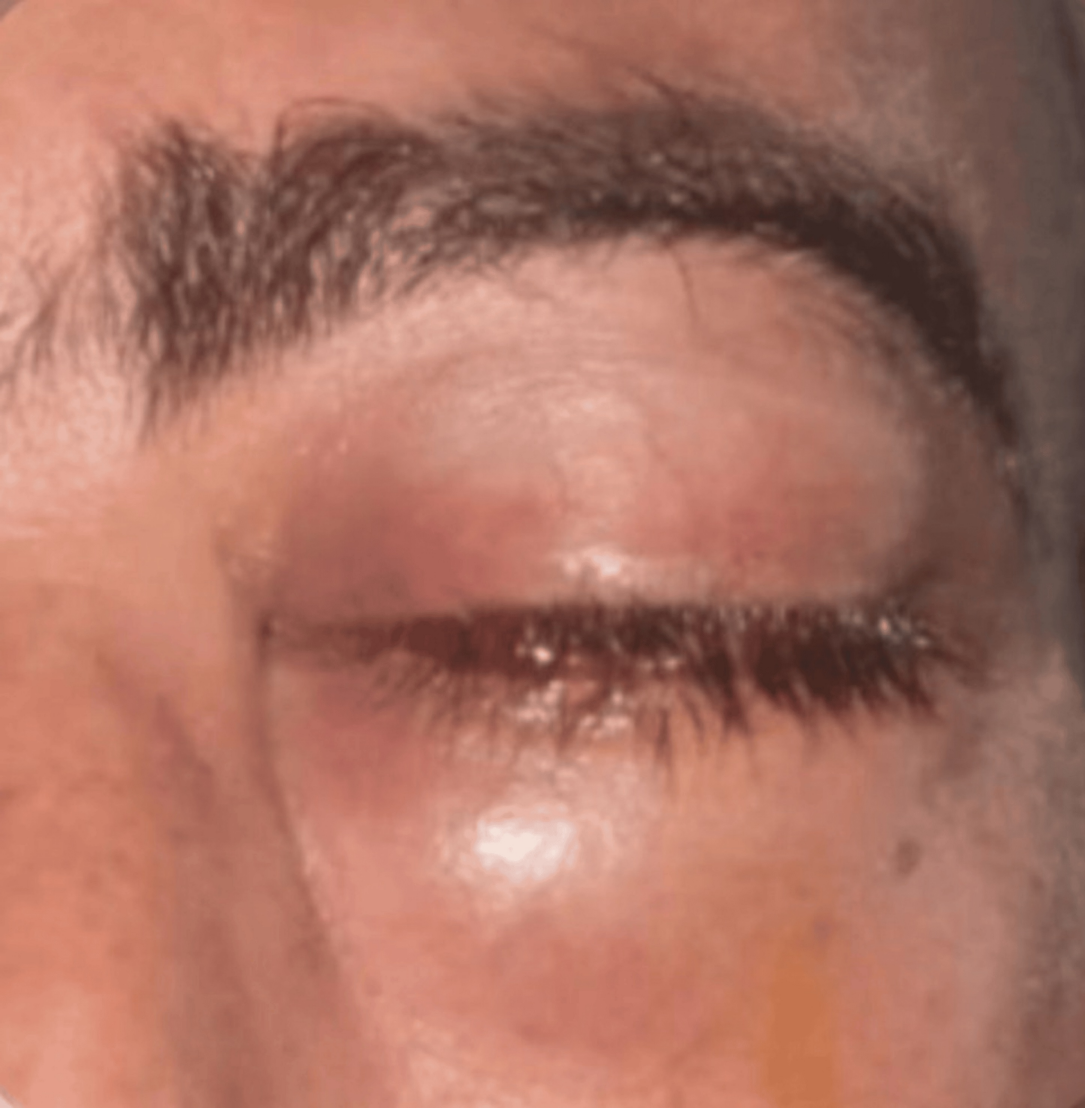
Photograph of the patient upon admission Significant periorbital edema associated with exophthalmos Image courtesy: Written informed consent to publish this article has been obtained from the patient

**Figure 2:**
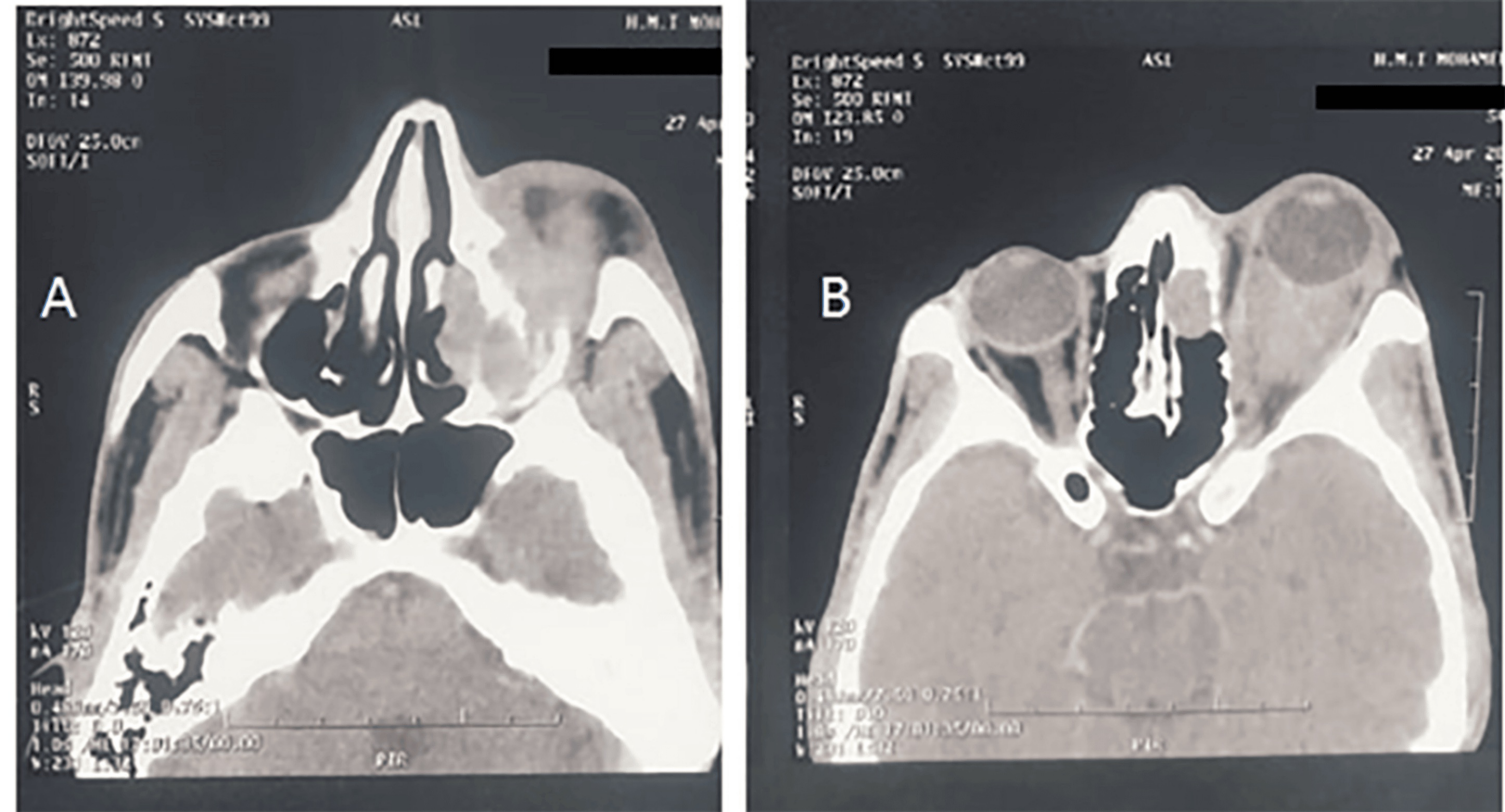
CT scan (axial slices) Presence of a roughly oval, well-defined, hypodense formation with thickened walls in the left orbit, measuring 20 x 16 mm, suggestive of an orbital abscess. This formation exerts a mass effect on the oculomotor muscles, causing grade 2 exophthalmos. Infiltration of the soft tissues and intra- and extraconal fat. This is associated with complete filling of the left maxillary sinus, frontal sinus, and lacrimal-nasal canal The image is suggestive of orbital cellulitis complicated by an orbital abscess, classified as Chandler stage IV

Examination of the facial skeleton revealed significant hyperalgesic upper and lower left eyelid edema with associated exophthalmos and chemosis. Ophthalmological examination revealed severely reduced visual acuity with ophthalmoplegia and increased ocular pressure. Examination of the lymph nodes revealed bilateral cervical lymphadenopathy measuring approximately 3 cm in diameter (Figure 1). A CT scan of the maxillofacial (MF) region was requested and revealed orbital cellulitis with a Chandler stage 4 orbital abscess (Figure 2).

**Figure 3:**
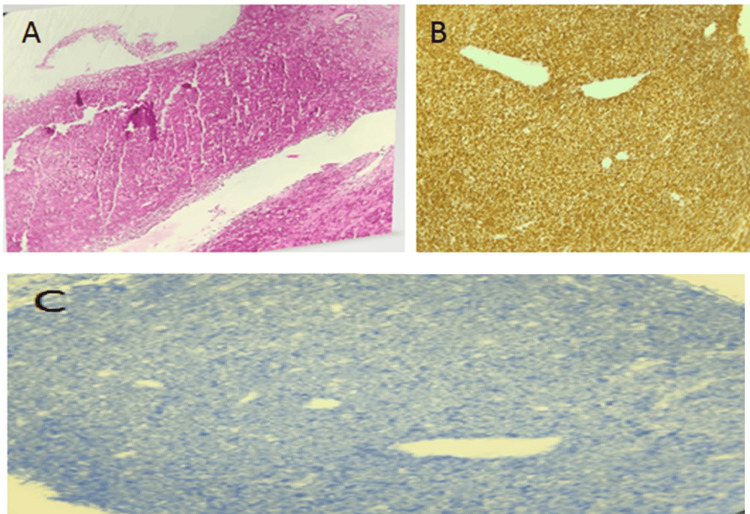
Histological images Diffuse lymphoid tumor proliferation 40× H&E (A). 100% Ki67 positivity of tumor cells, 40× (B). Tumor proliferation negative for Bcl2, 40× (C) HE: hematoxylin and eosin

**Figure 4:**
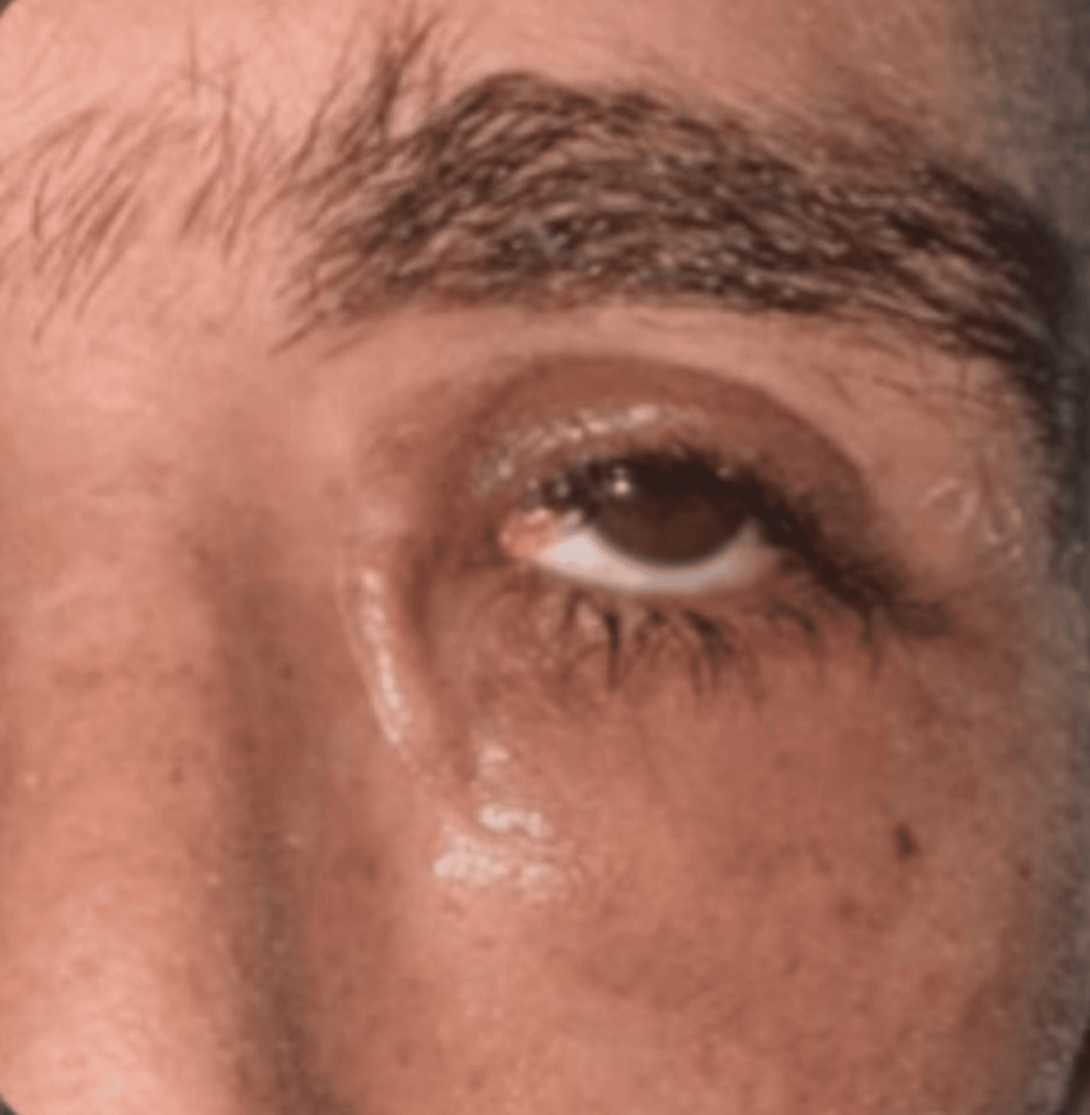
Photograph of the patient on the 22nd day of treatment Remission of left orbital edema Image courtesy: Written informed consent to publish this article has been obtained from the patient

At this stage, the diagnosis was orbital cellulitis with collection, and the patient was admitted to the operating room, where he underwent paracanthal exploration of the left orbital cavity and an endosinus biopsy, which confirmed Burkitt's lymphoma (Figures 3A-3C). The patient was referred to the clinical hematology department for treatment, where he underwent a therapeutic assessment and received the rituximab plus cyclophosphamide, doxorubicin, vincristine, and dexamethasone (R-CODOX-M) protocol. On day 22, there was a significant clinical improvement with complete regression of the orbital swelling and a decrease in visual acuity to 2/10 (Figure 4).

## Discussion

Orbital localization occurs in approximately 20% of cases. However, it should be considered when acute unilateral exophthalmos appears in young adults. Ocular involvement in malignant hematological disorders may be indicative of the disease, in which case an assessment of its extent is required to guide treatment and follow-up examinations [1].

BL has been described in HIV infection. This form occurs at a degree of immunodeficiency with an average CD4 lymphocyte count greater than 200/mm^3^. The presentation is similar to the sporadic form [1].

In Morocco, the incidence of BL remains unknown. Its epidemiological profile is more consistent with sporadic lymphoma. In a study conducted by Madani et al. [2] in 2005, maxillary localization accounted for 9.5% of cases, with abdominal involvement in 73.5% of cases. Otmani et al. in 2008 found oral involvement in 8% of 452 cases of NHL for BL [3].

Segbena et al. reported on 28 patients with MF localization: 39.3% of patients had upper jaw involvement, 28.6% had mandibular involvement, and 17.9% had involvement of both jaws from the outset. Eye and orbital involvement was noted in 39.3% of patients in Togo in 1992 [4].

The histopathological diagnosis is based on a biopsy of a lymph node or organ that may be affected, such as the bone marrow. In rare cases, laparoscopy may be performed for both diagnosis and treatment [5]. The histological appearance reveals small- to medium-sized cells with regular nuclei and immature reticular chromatin, containing a few nucleoli, often in a central position. There is significant basophilia of the cytoplasm, with a typical “starry sky” appearance, caused by the clarity of the reactive macrophages dispersed within a dense, basophilic tumor population [6]. Immunophenotyping completes the diagnosis by identifying the presence of B markers. Cytogenetic testing for chromosomal abnormalities and Ig gene rearrangements usefully complements the histological examination. It can only be performed on a fresh or frozen biopsy specimen in a specialized laboratory.

The differential diagnosis is made with an infectious process of dental origin, with benign tumor pathology when BL is unilateral. On the other hand, bilateral involvement strongly suggests a tumor origin. BL is an emergency. The therapeutic strategy is clearly defined by regularly updated protocols aimed at achieving maximum efficacy with minimal toxicity. The priority is to perform a rapid and complete assessment of the extent of the tumor, which is an essential criterion for prognosis and therapeutic choice [6].

Treatment must be initiated quickly because these tumors grow rapidly. Intensive alternating polychemotherapy with cyclophosphamide, vincristine, doxorubicin, methotrexate, ifosfamide, etoposide, cytarabine (CODOX-M/IVAC) plus rituximab is effective in children and adults less than 60 years of age. In selected patients less than 60 years of age and many patients greater than 60 years of age, regimens such as rituximab plus etoposide, prednisone, vincristine (Oncovin), and doxorubicin (at adjusted rituximab, etoposide, prednisone, vincristine, cyclophosphamide, and doxorubicin doses) are also commonly used with success. In patients without CNS involvement, CNS prophylaxis (e.g., with systemic and/or intrathecal methotrexate and/or cytarabine) is essential [6].

Our patient, who received the R-CODOX-M protocol, is currently in complete remission at day 22 of follow-up. Kissi et al. had complete remission at two years of follow-up [6]. Segbena et al. had complete remission at 46 days in 32.1% of patients with chemotherapy based on cyclophosphamide, chlorambucil, methotrexate, and vincristine [4].

## Conclusions

BL is a highly malignant cancer mainly observed in cases of HIV infection. Orbital localization is rare, and diagnosis is based on biopsy of samples from the affected organs or lymph node puncture. Treatment must be initiated quickly and involves intensive polychemotherapy, which allows for remission within a short period of time.
